# Long-Term Outcomes of Surgical and Transcatheter Interventions for Tricuspid Regurgitation: A Comprehensive Review

**DOI:** 10.3390/jcm14072451

**Published:** 2025-04-03

**Authors:** Vasiliki Tasouli-Drakou, Ibrahim Youssef, Arsalan Siddiqui, Tahir Tak

**Affiliations:** 1 Department of Internal Medicine, Kirk Kerkorian School of Medicine at the University of Nevada, Las Vegas, NV 89106, USA; arsalan.siddiqui@unlv.edu (A.S.); tahir.tak@va.gov (T.T.); 2 Department of Internal Medicine, Valley Health System, Las Vegas, NV 89118, USA; ibrahim.youssef@uhsinc.com

**Keywords:** tricuspid regurgitation, tricuspid valve repair, transcatheter devices, percutaneous tricuspid surgery, tricuspid valve treatment

## Abstract

Impacting more than 70 million people worldwide, tricuspid regurgitation (TR) refers to the retrograde flow of blood from the right ventricle to the right atrium due to the improper closure of the tricuspid valve. Depending on the severity of TR, signs and symptoms can range from asymptomatic to features of right heart failure, including dyspnea, exercise intolerance, peripheral edema, and ascites. Severe features such as these necessitate treatment. In recent years, advancements in management, including surgical and transcatheter interventions, have taken prominence, leading to improved short-term outcomes in this patient population. However, there is still a dearth of evidence regarding the long-term outcomes of surgical and transcatheter interventions for TR. This comprehensive review aims to present clinicians with recent findings from pivotal clinical studies on interventional clinical outcomes in an effort to help guide their judgment when it comes to deciding the best course of treatment for their patients.

## 1. Introduction

In normal cardiac anatomy, the tricuspid valve (TV) is composed of the septal, anterior, and posterior leaflets, with the posterior attached to the tricuspid annulus, a saddle-shaped ring of tissue situated right above the interventricular septum [1]. It is responsible for conducting blood flow between the right atrium (RA) and the right ventricle (RV) prior to it reaching the lungs [2]. Multiple conditions can affect the structure and function of the valve, leading to conditions such as tricuspid stenosis and tricuspid regurgitation (TR). TR affects more than 70 million people worldwide, being more prevalent in women [3]. However, 80% to 90% of asymptomatic individuals have been found to have physiologic TR on transthoracic echocardiography (TTE); thus, its presence in itself is not representative of pathology, and clinical correlation is required [4]. When it does manifest, however, signs and symptoms can range from asymptomatic to features of right-sided heart failure (HF), including dyspnea, exercise intolerance, jugular venous distension, peripheral edema, and ascites [5]. In this context, TTE is a crucial tool, allowing clinicians to assess TV morphology and function, the size and compliance of the RA, and the size and function of the right RV, as well as prognostic indices like RV systolic pressure and left-sided valve disease [6].

For many patients with severe TR, surgical TV repair and replacement were some of the mainstream options for treatment in the setting of ineffective medical management or those with concomitant left-sided heart surgery indications. In recent years, however, transcatheter devices for the treatment of TR have emerged in an effort to reduce the morbidity and mortality in patients with severe TR, especially for those who are deemed high-risk surgical candidates [7]. With the long-term outcomes of emerging new transcatheter systems coming to surface, an increasing number of retrospective studies and clinical trials comparing these interventions to traditional treatment, as well as amongst each other has started to become available. Given this, our review aims to compile and provide a comprehensive scoping review on the clinical outcomes of both surgical and transcatheter interventions for the management of severe TR. Additionally, it aims to provide clinicians with an organized collection of information that could help guide their judgment when it comes to deciding the best course of treatment for their patients.

## 2. Materials and Methods

A literature search was conducted across multiple bibliographic databases, including PubMed, MEDLINE, and Google Scholar. Medical Subject Headings (MeSH) terms used in the search included “tricuspid valve insufficiency”, “cardiac valve annuloplasty”, “cardiac catheterization”, “heart valve prosthesis implantation”, “treatment outcome”. Boolean operators “AND”, “OR” were used to combine these terms for search results. The primary questions to answer for this comprehensive review were “What are the currently available surgical and transcatheter interventions for the treatment of severe tricuspid regurgitations?” and “What are their reported outcomes?”. Given that the most recent 2020 guidelines published in conjunction by the American Heart Association (AHA) and the American College of Cardiology (ACC) were published in December 2020, the date range was adjusted to include the relevant literature published thereafter [8]. Article types included clinical trials, randomized controlled trials, literature reviews, systematic reviews, and meta-analyses. While an emphasis was placed on including the most recent and relevant publications, certain publications from outside the date range were also included to add to the comprehensive character of this review article.

## 3. TR Classifications and Echocardiographic Criteria for TR Grading

TR is divided into primary and secondary types. Primary TR is caused by an anatomical abnormality of the TV apparatus, and accounts for 8 to 10% of cases [9]. The causes of primary TR can include rheumatic fever, in which tricuspid leaflets become thickened and calcified, as well as Ebstein anomaly, in which there is apical displacement of the septal and posterior leaflets. Other prominent primary TR causes include infective endocarditis, degenerative disease such as TV prolapse, and iatrogenic causes including radiation therapy and medications like fenfluramine–phentermine [10,11].

Secondary TR is more prevalent and is attributed to maladaptive RV or RA remodeling leading to the annular dilation of TV and leaflet tethering. It can be further classified into atrial secondary TR (A-STR), which is often associated with RA enlargement and atrial fibrillation (AF), and ventricular secondary TR (V-STR), which is associated with RV enlargement and pulmonary hypertension [12]. Moderate or severe TR, especially secondary TR, can progress to adverse clinical outcomes and lead to irreversible myocardial damage if left untreated [13]. Additionally, increased mortality and morbidity in cases of secondary TR are associated with the presence of risk factors such as older age, chronic kidney disease (CKD), and hypertension [14].

The diagnosis of A-STR occurs when six criteria are met. These include clinically relevant secondary TR, predominant TA dilation, predominant RA dilation with increased end-systolic RA:RV ratio, an absence of significant tricuspid leaflet tethering, RV conical remodeling with the predominant enlargement of the RV basal dimension, and preserved LV and RV systolic function [15]. While the prevalence of A-STR is lower than that of V-STR, a study by Gavazzoni et al. found that A-STR patients had a smaller tenting height (TH), a larger end-diastolic tricuspid annulus area and better RV longitudinal function compared to V-STR patients, while V-STR patients were at a greater risk for all-cause death and hospitalization for heart failure at the 1-year endpoint [16]. Another study by Russo et al. comparing A-STR and V-STR patients who underwent tricuspid transcatheter edge-to-edge repair (T-TEER) found that A-STR was independently associated with increased survival at a median 10-month follow-up compared to V-STR patients despite the similar procedural success rates [17]. These findings suggest that A-STR patients could be viewed as a separate subgroup from V-STR patients due to the possibly different underlying mechanisms and differences in the geometry of the right heart structures between patients with A-STR and V-STR [16].

The classification of TR is often based on the severity of the clinical and echocardiographic findings. The 2020 AHA/ACC guidelines for the management of patients with valvular heart disease classifies TR into three stages: stage B is defined as progressive TR with no hemodynamic and clinical symptoms, stage C is defined as asymptomatic severe TR, and stage D is defined as symptomatic severe TR [8]. Unlike stage B, stages C and D are characterized by a central jet greater than or equal to 50% of RA, a vena contracta (VC) width greater than or equal to 0.7 cm, an effective regurgitant orifice greater than or equal to 0.4 cm^2^, a regurgitant volume of greater than or equal to 45 mL, dilated RV and RA, and elevated venous pressure. Stage D patients further exhibit dyspnea on exertion, fatigue, ascites, and edema [8]. Another proposed classification system based on leaflet mobility, known as the Carpentier classification system, exists. According to this system, TR is classified into four types: type I is characterized by annular dilation and type II is characterized by leaflet prolapse, while types IIIa and IIIb exhibit degenerative leaflet changes and tenting, respectively, with restricted motion in systole [11,18].

**Figure 1:**
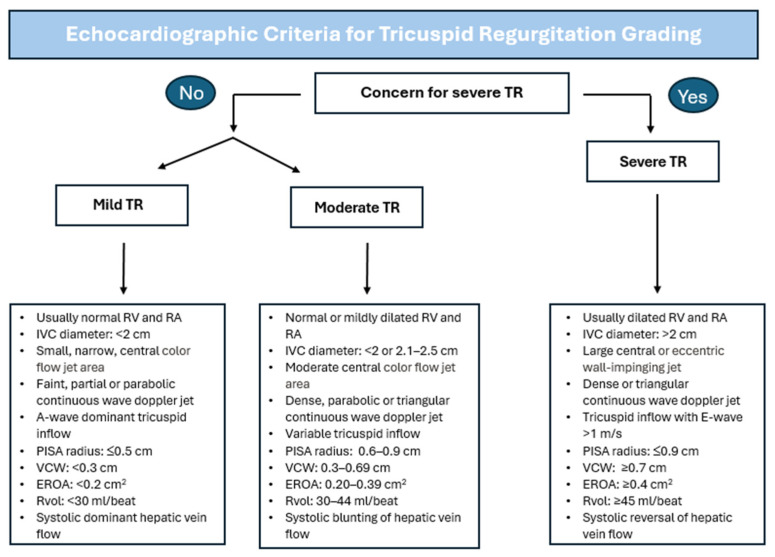
Figure summarizing the echocardiographic criteria for the grading of tricuspid regurgitation as mild, moderate, and severe using echocardiography according to the 2024 AHA and the 2017 ASE Guidelines [19,20]. TR: Tricuspid Regurgitation; RV: Right Ventricle; RA: Right Atrium; IVC: Inferior Vena Cava; VCW: Vena Contracta Width; PISA: Proximal Isovelocity Surface Area; EROA: Effective Regurgitant Orifice Area; Rvol: Regurgitant Volume.

The classification of TR is essential for identifying patients who could benefit from surgical/percutaneous interventions, as discussed below. However, given the progressive nature of the disease, it has been proposed that TR should be further stratified based on echocardiographic criteria. In 2024, the AHA published parameters for grading TR based on structural, qualitative, semiquantitative, and quantitative criteria [19]. These parameters are based on the guidelines issued by the American Society of Echocardiography (ASE) [20]. In this framework, mild TR is defined by a VC width of less than 3 mm, and an effective regurgitant orifice area (EROA) of less than 20 mm^2^ [21,22]. Moderate TR is characterized by a VC width of 3–6.9 mm and an EROA of 20–39 mm^2^. The primary distinction in this proposed grading is the further stratification of severe TR into three categories: severe, massive, and torrential TR. Severe TR is defined by a VC width of 7–13 mm, an EROA of 40–59 mm^2^, and a three-dimensional VC area or Quantitative Doppler (QD) EROA of 75–94 mm^2^. Massive corresponds to a VC 14–20 mm, EROA 60–79 mm^2^, and three-dimensional EROA or QD EROA of 95–114 mm^2^. Lastly, torrential TR is defined by a VC greater than 21 mm, EROA greater than 80 mm^2^, and three-dimensional EROA or QD EROA greater than 115 mm^2^. Further stratifying TR severity could lead to a more precise and quantitative assessment of the efficacy and impact interventions may provide [21]. Figure 1 summarizes the AHA and ASE guidelines for the TR grading of mild, moderate, and severe TR. The assessment and evaluation of TV function is not only limited to echocardiography findings but also requires clinical correlation, including medical history and a physical evaluation, as patients may present with dyspnea, fatigue, edema in the lower extremities, and hepatic congestion [3]. The latter two symptoms may also signify the presence of right-sided HF.

## 4. Approaches to TR Management and Current State-of-the-Art

### 4.1. Indications for TV Surgery

In 2020, the AHA issued guidelines on the management of patients with valvular heart diseases, including those with TR. In patients with right-sided HF attributed to severe TR, diuretics may be useful, while if TR is confirmed to be secondary in nature, then cardiologists will need to treat the underlying conditions [8]. This includes rhythm control for AF, Goal-Directed Medical Therapy (GDMT) for HF with a reduced left ventricular ejection fraction (LVEF), and pulmonary vasodilators for a reduction in pulmonary arterial pressures.

Surgical interventions for TR have been in use since 1961 [23]. They are divided into repair and replacement techniques, which will be discussed further below. Currently, AHA guidelines recommend tricuspid surgery for patients with severe TR undergoing concomitant left-sided cardiac surgery [24]. However, there are a few instances where isolated TV surgery may be beneficial. These instances include patients with symptoms of right-sided HF and isolated primary TR, those with right-sided HF and severe isolated secondary TR attributable to annular dilation, patients with severe primary TR and progressive RV dilation or systolic dysfunction, and individuals with right-sided HF and severe TR who have undergone previous left-sided valve surgery and do not have severe pulmonary hypertension or severe RV systolic dysfunction [8].

**Figure 2:**
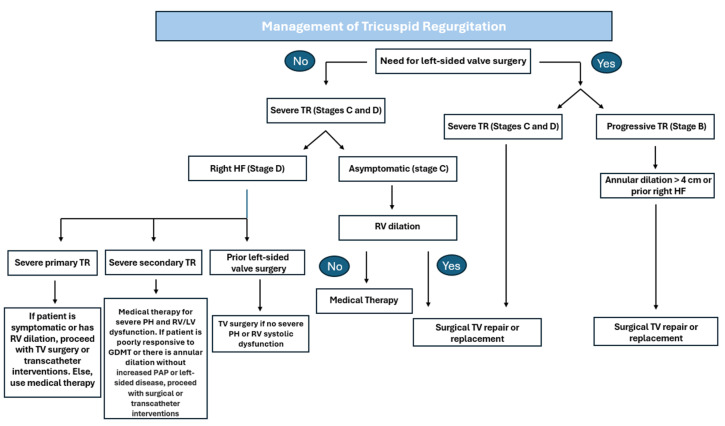
Algorithm for approaching TR management based on AHA and ESC guidelines [8,25]. TR: Tricuspid Regurgitation; HF: Heart Failure; TV: Tricuspid Valve; RV: Right Ventricle; LV: Left Ventricle; PAP: Pulmonary Artery Pressure; PH: Pulmonary Hypertension.

Consistent with the AHA guidelines, the current 2021 European Society of Cardiology (ESC) guidelines recommend surgery in symptomatic patients with severe primary TR, while they also state that TV repair should be performed liberally during left-sided surgery in patients with secondary TR [25]. Additionally, like the AHA guidelines, the ESC guidelines support medical therapy for patients with right-sided HF (i.e., diuretics) or pulmonary hypertension. Nonetheless, when comparing the algorithms proposed by the European and American guidelines, the AHA guidelines do not list transcatheter interventions. The ESC algorithm for TR management lists transcatheter therapy as an option for symptomatic patients with severe secondary TR and no RV dysfunction and for candidates who are not eligible for surgery. Another discrepancy is that the AHA guidelines mention the need for TV surgery for patients with severe secondary TR caused by annular dilation in the absence of pulmonary hypertension or left-sided disease [26]. An algorithm summarizing the approaches to TR management based on the AHA and ESC guidelines is shown in Figure 2.

### 4.2. Surgical Repair and Replacement Techniques

**Figure 3:**
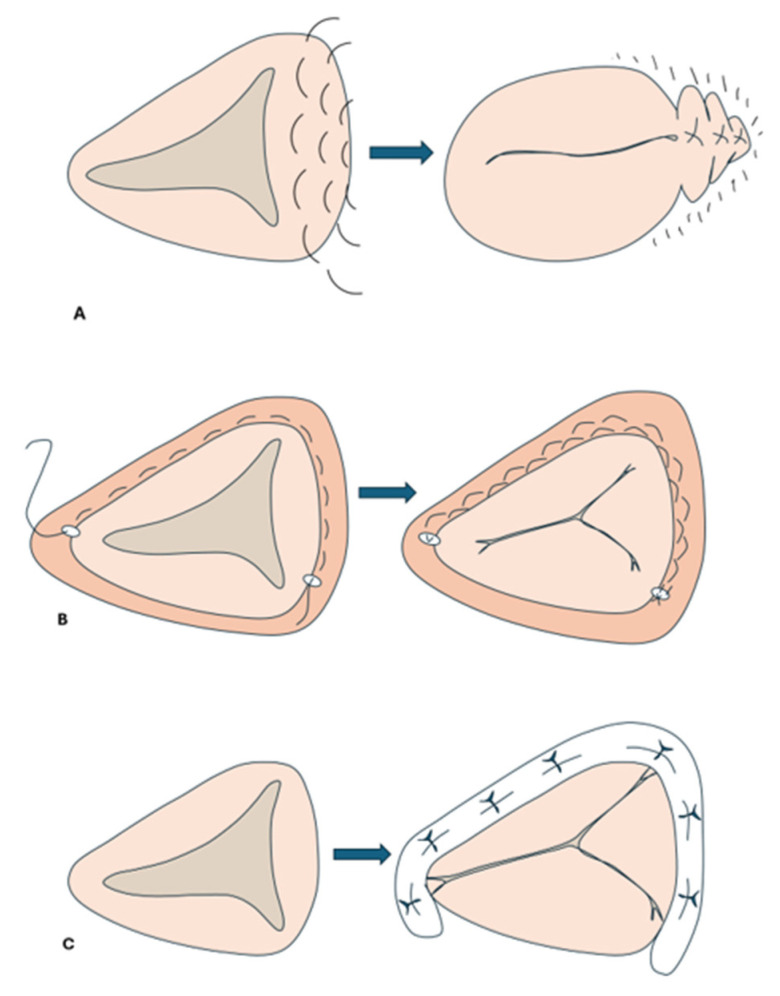
Infographic Demonstrating the Kay (**A**), De Vega (**B**), and Ring (**C**) Annuloplasty techniques.

Surgical repair techniques are divided into three main categories: annuloplasty, leaflet repair techniques, and others [27]. Annuloplasty techniques are separated into suture and ring techniques. Two great examples of suture annuloplasty techniques are the Kay and De Vega procedures (Figure 3). The former involves a figure-of-eight suture plication of the posterior leaflet while the latter involves reducing the area of the tricuspid annulus with two parallel sutures around the perimeter of the orifice [28]. Given that, in secondary TR, the tricuspid annulus is dilated and flattened, the use of an annular ring may also aim to downsize the tricuspid annulus [29]. Ring devices differ in size, shape, and stiffness. These rings are open at the level of the triangle of Koch to avoid atrioventricular (AV) block [28]. A study by Tang et al. found that although ring annuloplasty was associated with better long-term survival compared to suture annuloplasty, both groups exhibited significant TR recurrence, reaching up to 20–30% for patients who received ring annuloplasty and 60–70% for suture annuloplasty patients [30].

Surgical tricuspid leaflet repair techniques include anterior leaflet augmentation, which is performed by making an incision at the base of the anterior leaflet between the anteroseptal and anteroposterior commissures via the placement of an annuloplasty ring, and the “clover” technique, in which the midpoints of the TV leaflets are stitched together [31]. Other techniques include suture bicuspidization, in which a double pledged-supported mattress suture is placed from the anteroposterior to the anteroseptal commissure along the posterior annulus, and artificial polytetrafluoroethylene sutures are used to treat prolapse of the A1-A2 portion of the TV anterior leaflet [28,31].

When TV repair is not feasible, when there is a need for concurrent left-sided heart surgery, or when repair has failed, surgical replacement may be considered using a biological or mechanical prosthesis [32]. Although mechanical prostheses are considered to be more durable than biological ones, which tend to be porcine, they are associated with a greater risk of valve thrombosis, thromboembolism, and bleeding. Moreover, 30-day mortality rates in isolated TV replacement have been reported by single-centered studies to reach as high as 14.8% and 17.6% [33]. Due to the poor short-term outcomes of both surgical repair and replacement, a shift towards transcatheter interventions is taking place.

### 4.3. Transcatheter Tricuspid Valve Repair

Transcather Triscupid Valve Repair (TTVr) is a minimally invasive procedure indicated for severe TR in patients deemed to be at high surgical risk as it is associated with fewer perioperative complications than surgical TV intervention [34]. Anatomical suitability is an important factor when determining eligibility for TTVr. Candidates for this procedure include those who have a pure annular dilation with coaptation defect of ≤10 mm and no severe leaflet tethering, as the goal of this procedure is to preserve the native valve and perform annuloplasty or to correct the coaptation of the leaflets [24,35]. Given this, TTVr may be considered a more suitable option than transcatheter TV replacement (TTVR) for patients with RV dysfunction, especially those with contraindications to using anticoagulation, and patients with damage to adjacent heart structures [35].

**Figure 4:**
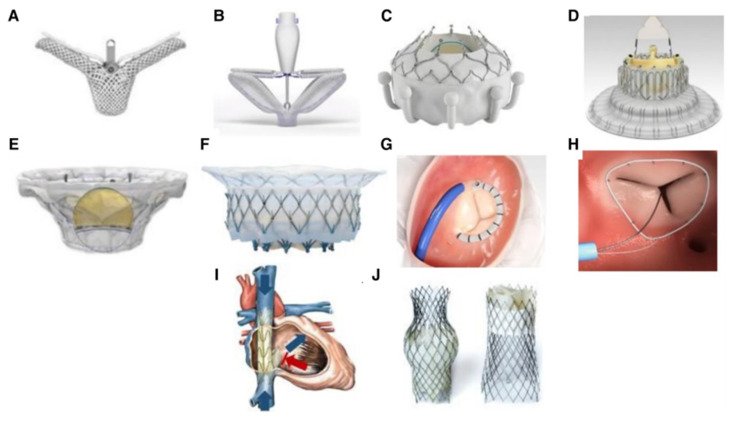
Figure illustrating the different devices for tricuspid regurgitation. (**A**) TriClip (Abbott Vascular, Santa Clara, CA, USA); (**B**) PASCAL system (Edwards Lifesciences, Irvine, CA, USA); (**C**) EVOQUE system (Edwards Lifesciences, Irvine, CA, USA); (**D**) LuX-Valve (Jenscare Biotechnology Co., Ningbo, China); (**E**) cardiovalve (Boston Medical, Shrewsbury, MA, USA); (**F**) Intrepid valve (Medtronic Plc, Minneapolis, MN, USA); (**G**) cardioband tricuspid valve reconstruction system (Edwards Lifesciences, Irvine, CA, USA); (**H**) tri-Ring annuloplasty system (cardiac implants, California, USA; (**I**) TRICENTO system (Medira AG, Balingen, Germany); (**J**) TricValve (NVT, Muri, Switzerland). Reproduction allowed under the terms of the Creative Commons Attributions License https://creativecommons.org/licenses/by/4.0/ (Accessed on 1 April 2025) [45].

Multiple methods and devices have been invented for TTVr. In April 2024, the Abbott TriClip G4 Delivery System became the first Food and Drug Administration (FDA) approved device for transcatheter edge-to-edge (TEER) repair of the TV for moderate to severe TR (Figure 4) [36]. The TriClip Delivery System is a coaptation device, bringing leaflets along the anterior–posterior or posterior–septal locations closer [24]. There are four different implant sizes which include a clip delivery system (CDS) and a steerable guide catheter (SGC) to guide and advance an implantable clip with grippers [37]. The TriClip was designed to replace the “off-label” use of nonspecific edge-to-edge devices such as the MitraClip for TTVR [38]. It was created to accommodate the larger coaptation gaps and sizes of the TV leaflets, and contains an additional SGC knob for better steering and advancement into the RA [39]. Furthermore, in a study by Balata et al., TriClip demonstrated greater efficacy in reducing vena contracta compared to MitraClip [40].

Similarly, PASCAL and PASCAL Ace are also used in TEER TTVr techniques. These devices involve paddles with clasps to approximate the leaflets of the TV, and use a central spacer to decrease the regurgitation area [41]. PASCAL Ace is narrower than PASCAL, allowing for it to be advanced through more complex structures, while both devices are able to detach from their steerable catheter distally after their desirable placement, allowing for more than one of them to be used if necessary. They are placed under general anesthesia, with fluoroscopic and transesophageal echocardiography guidance.

In comparison to TEER, which works on the leaflets of the TV, transcatheter annuloplasty techniques can also be used. Cardioband is one of these techniques. The implant of this device is a contractible wire covered by a polyester fiber delivered using a steerable catheter, and it becomes fixed to the annulus of the valve with a series of stainless-steel anchors [42]. After this, the wire is contracted under fluoroscopic and echocardiographic guidance until the desired annulus reduction for TR management is achieved. K-clip is another novel annuloplasty technique. The device includes a clamp made of an anchor and a clip that bites into the annulus of the TV and folds it with a corkscrew to reduce its circumference and improve the coaptation of the valve leaflets [43]. Four clamp sizes are currently available. Additionally, Mistral is a technique that targets the chordae tendineae of the TV rather than the annulus or the leaflets to improve coaptation [44]. This involves the implantation of a spiral-shaped wire rotated around the chordae tendineae under echocardiographic guidance until the desired level is reached.

### 4.4. Transcatheter Tricuspid Valve Replacement

Although TTVr is generally considered to be a safe option for the treatment of TR, there are certain instances during which its limited use might be suggested. As stated above, there are certain characteristics favoring transcatheter TV replacement (TTVR) versus TTVr, including the presence of a large coaptation gap (>10 mm) and tricuspid annulus (up to 55 mm), as well as the presence of tethered leaflets and permanent pacemaker (PPM) lead-induced TR [46]. For this reason, there has been an increased interest in the development of devices for TTVR.

In February 2024, the EVOQUE Tricuspid Valve Replacement System became the first FDA approved TTVR [36]. The EVOQUE device system, whose outer diameter is 28-Fr, contains bovine pericardial leaflets and a self-expanding nitinol frame, allowing the device to flex in the anterior–posterior and septal–lateral planes and to conform to the valve anatomy [47]. Its use is indicated in patients with demonstrated right-sided HF, New York Heart Association (NYHA) functional class II-IV symptoms with the presence of peripheral edema or ascites despite the use of optimal diuretic therapy, and in patients who may be considered at high risk for surgery. Like all transcatheter TV devices, the EVOQUE device system is placed under real-time transesophageal echocardiography and fluoroscopic guidance, and advanced across the TV annulus.

While it was the first FDA-approved device, the EVOQUE system is not the only type of transcatheter replacement system for TR that has been tested. For example, the first-in-human implantations of TTVR systems, such as the MonarQ system and the LuX-Valve Plus systems, were reported in the literature in 2023. The MonarQ system, comprising two nitinol frames, polyester sealing skirts, and a central 30 mm diameter trileaflet bioprosthetic valve, is delivered through a transjugular 30 Fr system, while the LuX-Valve Plus system, delivered through a transjugular 33 Fr system, consists of a trileaflet prosthetic valve with treated bovine pericardium, a self-expanding nitinol valve stent, an interventricular septal anchoring component, and two polytetrafluoroethylene-covered graspers [48,49,50]. The LuX-Valve Plus is an upgrade of the LuX-Valve TTVR system. Other TTVR systems include the Topaz, whose design is similar to that of the MonarQ but which uses a transfemoral 29 Fr delivery system, and the Cardiovalve, whose design is similar to the LuX-Valve Plus system but which uses a 32 Fr steerable transfemoral catheter for delivery [51,52]. The NaviGate TTVR system encompasses a nitinol self-expanding conical stent with a trileaflet equine pericardial valve [52]. Its delivery sheath size is 42 Fr and its access can be transjugular or transatrial. It is therefore not unlikely that more types of TTVR systems with similar designs will become approved for use in the near future.

The aforementioned TTVR systems are orthotopic, in essence replacing the natural TV at the same position. However, heterotopic systems also exist that do not interfere with the natural valve. One such system is the TriValve system; it consists of two self-expanding biological valves implanted in the superior and inferior vena cava and was designed to reduce systemic venous congestion in patients with symptomatic right-sided HF due to severe TR, especially those with hepatic congestion [53]. The TRICENTO system also aims to reduce venous congestion by employing a self-expanding nitinol stent frame covered by thin porcine pericardium into the superior and inferior vena cavae [54]. This system may be deemed beneficial for symptomatic patients who may be considered ineligible for surgery or other transcatheter treatment systems due to TR characteristics. Finally, balloon-expandable valves such as Sapien 3 and Sapien XT for caval valve implantation have also been described in the literature [55].

Regardless of their design or method of employment, TTVR systems are not without drawbacks, as their more invasive nature is associated with an increased risk of bleeding and a need for anticoagulation [24]. Although the long-term outcomes will be discussed in further detail below, it is important to be cognizant of the fact that patient care and treatment should remain individualized, taking into consideration the patient’s anatomy and TR characteristics.

## 5. Long-Term Outcomes

### 5.1. Surgical Replacement Outcomes

As previously stated, surgical interventions are recommended for severe TR when performed with concomitant surgery for left-sided heart disease. A single-center study by Park et al., which compared patients that underwent surgical TV replacement (STVR) with surgical TV repair (STVr) after left-sided valve surgery for moderate–severe TR, found that although there were no significant differences in valve-related thrombosis, stroke, or mortality at 1, 5, and 10 years between the two groups, postoperative hospital stays were significantly prolonged in the STVR group [56]. Five- and ten-year survival rates were 93% and 63% for the STVR (mechanical or bioprosthesis) group, and 93% and 81% for the STVr group, the latter of which underwent either De Vega annuloplasty, prosthetic ring annuloplasty with a St. Jude Tailor Flexible Ring, or prosthetic ring annuloplasty with a Carpentier–Edwards semi-rigid ring. A newer study by Dai et al., which assessed 37 patients with moderate-to-severe TR who underwent a minimally invasive STVr using either a SOVERINGTM band or an Edwards MC3 annuloplasty ring after left-sided valve surgery, found that the in-hospital and 30-day mortality rates were 2.7%, with 10.8% of patients undergoing re-exploration for bleeding and 9.3% of patients having a recurrent TR classified as severe or worse [57]. A total of 72.2% of patients did show an improvement in the NYHA class. While this rate of reoperation for bleeding is similar to the one reported in a similar study by Azarnoush et al., the 30-day mortality rate in STVr patients who underwent annuloplasty with either a SOVERINGTM ring or a Bex linear reducer in this study reached as high as 11.7% [58].

Surgical interventions, however, may be considered for isolated severe primary or secondary TR. A retrospective cohort study of 537 patients who underwent isolated TV surgery, including TV annuloplasty and replacement, as well as non-annuloplasty TV repairs and valvotomies, found that in-hospital and total mortality were 7.4% and 39.3% during a mean follow-up of 4.82 years, with cardiovascular causes of death representing 45% of in-hospital deaths and 52% of post-discharge deaths [59]. End-of-study outcomes also revealed the rates for new-onset AF and readmission for decompensated congestive HF to be 27.4% and 29.2%, respectively, with 7.5% of subjects undergoing PPM implantation and 2.4% receiving an implantable cardioverter–defibrillator. A meta-analysis of 5316 patients by Scotti et al. that investigated the outcomes from isolated STVR found a rate of 10% for new PPM implantation, a rate of 12% for bleeding, and an overall operative mortality rate of 12% [60].

When comparing isolated surgical repair with replacement for TR, a 2024 study comparing isolated STVR and STVr among older patients in the United States found that subjects who were offered STVR were more likely to be suffering from a comorbid condition such as hypertension, HF, AF, CKD, cancer, and rheumatic TV disease, while they detected no statistically significant differences in the incidence of stroke and TV reoperations (fewer than ten events for each group), and the in-hospital (13.2% versus 12.8%) and all-cause mortality (40.2% versus 37.5%) at 3 years among the STVR and STVr groups [61]. They also found that STVR subjects were more likely to undergo postoperative PPM implantation. These findings are consistent with a prior international study by Russo et al. The study found no statistically significant difference in the 30-day mortality of the STVR (8%) and STVr (4%) subjects; however, it did showcase that patients in the STVR group were more likely to have higher rates for exploration for bleeding (13.1% vs. 6.9%), PPM implantation (12.0% versus 5.1%), and blood transfusion (62% versus 46%) than those in the isolated STVr subgroup [62]. Survival rates at 5 and 7 years were 75% and 56% for the TVr group and 66% and 58% for the TVR one.

Retrospective studies comparing the surgical and medical therapies for severe TR have also been published in recent years (Table 1). TRI-SCORE, a relatively new risk score model to predict the outcome of patients after isolated TV surgery for severe TR, is based on the presence of eight risk factors, including an age greater than 70, LVEF < 60%, moderate or severe RV dysfunction, NYHA functional class III/IV, right-sided HF signs, a daily furosemide dose equal to or greater than 125 mg, elevated total bilirubin, and a glomerular filtration rate of less than 30 mL/min [63]. A score less than or equal to 3 represents low surgical risk, while scores of 4–5 and scores equal to or greater than 6 represent intermediate and high surgical risks for mortality, respectively [64]. This score was used in the retrospective study by Dreyfus et al., whose findings are summarized in Table 1, along with the studies by Axtell and Wang et al.

As seen in the aforementioned studies, the mortality rates of surgical interventions for severe TR may not vary significantly among patients undergoing either surgical TV repair or replacement. Nonetheless, they remain high, as demonstrated by the relatively increased rates of adverse events, such as bleeding and new-onset AF. The potential need for PPM implantation due to the high risk of developing an AV block following an intraoperative injury to the AV node and the bundle of His is further illustrated in the study by Fu et al., in which the PPM rate for isolated STVR was found to be 23%, significantly higher than that of isolated STVr cases (3.7%) [68]. Moreover, the majority of the aforementioned studies did not reference or compare preoperative and postoperative NYHA classes. The shift from high-risk surgical interventions to a transcatheter approach for the treatment of moderate or severe TR could therefore aim to monitor and improve long-term clinical outcomes and reduce intraoperative complications.

### 5.2. TTVr Outcomes

Multiple studies have been conducted to evaluate the effectiveness and safety of different TTVr devices, with patients followed to observe long-term outcomes. The TRILUMINATE study was an international, multicenter, single-arm prospective clinical trial that studied the TriClip, and its final 3-year outcomes were published in December 2024 [69]. A total of 98 patients with moderate or more severe TR who were at high surgical risk were included in this study. The trial studied the effectiveness of the device, as measured by echocardiographic evidence of TR reduction, as well as clinical improvement and safety. It reported promising results regarding these aspects after 3 years following TTVr. However, a limitation of this study was that it was an uncontrolled trial, making it difficult to conclude whether these results were directly due to TriClip use or other confounders. Thus, the TRILUMINATE Pivotal study was conducted. This was also an international multicenter clinical trial but was randomized at a 1:1 ratio. The results for the 350 initially recruited patients were published in March 2023, while the 1-year results of a total of 572 patients (including the initial 350 and a subsequent 222) were published in January 2025 [70,71]. These patients had severe or more TR and had a moderate or greater surgical risk. The study found that 1 year after the procedure, although subjects experienced a clinical improvement which was statistically significant compared to the control group, mortality and the rate of hospitalization for heart failure were similar in both groups. Table 2 summarizes the 3-year outcomes of the TRILUMINATE study and the 1-year outcomes of the TRILUMINATE Pivotal trial.

As for the PASCAL system, CLASP TR, a single-arm prospective international multicenter clinical trial, studied this system. In May 2023, the 1-year results of this study were published. A total of 65 patients were enrolled, 45 of whom returned for a 1-year evaluation visit after treatment [41]. The study included patients with severe functional or degenerative TR, who were symptomatic despite medical therapy. The study found a clinical improvement and reduction in TR grade in the patients studied. More recently, the PASTE study, published in January 2025, was a multicenter retrospective and prospective observational cohort study that evaluated the effectiveness of the PASCAL system in 16 European centers. It included 1059 patients who underwent this procedure, 96% of whom had severe or worse TR prior to the procedure [72]. The majority of the patients studied experienced a reduction in TR grade and clinical improvement 1 year after the procedure.

The Edwards Cardioband Tricuspid Valve Reconstruction System Early Feasibility study was a multicenter single-arm trial to evaluate the efficacy and safety of the Cardioband system. The study enrolled 37 patients in seven centers in the United States of America with moderate or greater TR who were symptomatic despite optimal therapy, and its 1-year outcomes were published in October 2022. This study also showed both clinical improvements and echocardiographic improvements in TR grade in the studied patients [73]. Similarly, the TRI-REPAIR study evaluated the Edwards Cardioband system. The 2-year outcomes of this multicenter single-arm clinical trial conducted in the European Union were published in February 2021. It included 30 patients from eight European centers with moderate or greater symptomatic TR who had a high surgical risk or who were not candidates for surgery [74]. At year 2 after the procedure, this study also found a TR grade reduction and clinical improvement in the majority of patients.

The outcomes of the Mistral technique have been studied. In July 2023, the outcomes one year after a procedure using this technique were published (MATTERS and MATTERS II trial). The study was a multicenter, single-arm trial that included nine patients with severe or greater TR with a high surgical risk [75]. The study showed a TR grade reduction and clinical improvement at the 1-year mark after the procedure. There was no report of whether there were any hospitalizations after Mistral placement in any of the patients studied, but there were no deaths over the period of this study.

Similarly to other studies evaluating TTVr devices, TriStar, a multicenter single-arm trial conducted in China, studied the use of K-clip. In December 2024, the outcomes one year after the procedure were published [76]. A total of 96 patients were included in this study with symptomatic massive or greater secondary TR and intermediate or high surgical risk. The study showed a TR grade reduction and clinical improvement over the period of the study. Table 3 summarizes the results of these major clinical trials that evaluated TTVr devices.

It is important to note here that, in addition to the clinical outcomes suggested by the aforementioned studies, various predictors of procedural success based on transesophageal anatomy have been developed for TTVr. One of them is the leaflet-to-annulus index (LAI) proposed by Tanaka et al. This is defined as the ratio of sum of the anterior and septal tricuspid leaflet length in relation to the septolateral tricuspid annulus [77]. The authors found that patients with residual TR of severe or greater class after TEER exhibit not only a lower LAI compared to patients whose residual TR was classified as moderate or less, but also a greater risk for all-cause mortality and heart failure hospitalization within one year after TEER. A similar index has been suggested by Pizzino et al. In their study, the authors proposed an anterior–posterior leaflet-to-annulus index (AP-LAI) [78]. Similarly to the study by Tanaka et al., Pizzino et al. found that patients with a moderate or greater residual TR after TEER also exhibited a lower AP-LAI compared to patients with less severe residual TR. They suggested that a reason for this observation is that a shorter leaflet length, and thus a lower AP-LAI, indicates that the chances of a successful clip insertion and TR reduction are fewer, with clinicians being forced to place the TEER devices farther away from the main TR jet.

Similarly to the idea of the TRI-SCORE, which is used in surgical interventions, a risk stratification score was proposed by Russo et al. to assist in patient selection and predict 12-month mortality and rehospitalization in those undergoing TTVr. This risk stratification system is known as the TRIVALVE score, basing its name on the International Multisite Transcatheter Tricuspid Valve Therapies (TRIVALVE) Registry [79]. With five parameters receiving a point score, including AF at baseline, a glomerular filtration rate of less than 30 mL/min, elevated bilirubin levels, signs of right HF, and an LVEF less than 50%, a cutoff value of 2.5 demonstrated 65.4% sensitivity and 60.5% specificity in predicting 12-month mortality or rehospitalization. It will thus be interesting to see how the implementation of such a score could influence the clinical outcomes of up-and-coming TTVr devices.

The comparison between surgical and transcatheter repair techniques has been a topic of growing importance in interventional cardiology now that multiple TTVr devices exist and evolving data from their usage have been published. Direct comparison studies are limited, largely due to the novel nature of TTVr. In November 2024, a study comparing tricuspid TEER with surgical TVr (STVr) was published. Of a total of 1143 patients, 409 underwent tricuspid TEER, and 734 underwent STVr [80]. The majority of TTVr patients in this study underwent TEER with the off-label use of MitralClip. A smaller number of patients in this study underwent TTVr using TriClip or Pascal systems. The study mainly found no difference in mortality or hospitalization due to HF rate difference between the TTVr and STVr groups at 2 years after the procedure. Although TTVr patients had lower in-hospital mortality (HR 0.84 95% CI 0.63–1.13, *p* = 0.25) and lower rates of PPM placement (*p* < 0.001) than STVr patients, the re-intervention rate in TTVr patients was higher than that of STVr patients (HR 8.03 95% CI 2.87–22.48, *p* < 0.001). A larger scale meta-analysis by Saito et al. was published in December 2024 comparing the outcomes of medical therapy, TTVr, and surgery. Regarding TTVr and surgery, the study demonstrated similar greater-than-1-year long-term mortality for both TTVr and surgical patients, but it also showed that TTVr patients were associated with a lower 30-day short-term mortality and periprocedural complications such as PPM placement [81]. Further clinical trials that control for confounders and directly compare TTVr and STVr can be conducted in the future for further analysis of the outcomes of the various TV repair techniques.

### 5.3. TTVR Outcomes

A 2022 meta-analysis by Bugan et al. that evaluated orthotopic TTVR placement in 321 patients with at least moderate native TR (95% of them had severe, massive, or torrential TR), found that TTVR outcomes were associated with a significant improvement in NYHA functional class and 6 min walking distance [82]. Other notable findings included the significantly reduced prevalence of severe or greater TR after TTVR, and reductions in RV end-diastolic basal diameter. Patients with the NaviGate, Lux-Valve, and EVOQUE TTVR systems were included. These findings are consistent with individual case studies and combined reports for these systems. For example, the published 2-year outcomes for the EVOQUE system not only demonstrated a 71% survival rate and significant improvement in symptoms of right-sided HF, such as ascites and peripheral edema, but also showed a significant reduction in TR severity, with 89% of surviving patients demonstrating a TR classification of mild or resolved at the follow-up (median duration of 520 days) [83]. A total of 20% of subjects demonstrated a NYHA classification of III or greater at follow-up, a reduction from 92% of patients prior to the initiation of treatment. Bleeding incidents were reported in 11% of subjects. Likewise, the 1-year outcomes for the LuX-Valve system in a study of 15 patients demonstrated a significant improvement in RV volume and NYHA functional class, with TR reducing to none/trace in 85.7% of subjects [84]. Adverse events were reported in a single patient, who was hospitalized for device thrombosis.

Although there are currently no trials comparing heterotopic TTVR systems, the 1-year results evaluating the TricValve and TRICENTO systems have reported positive results (Table 4). The TricValve study included 44 subjects with a mean age of 76.2 years, 82% of whom were women and all of whom had a NYHA functional class III/IV at baseline [85]. Similarly, the TRICENTO study included 21 patients with a mean age of 76 years, 67% of whom were women and 95% of whom had a NYHA functional class III/IV at baseline [54].

Currently, there are no publications that compare TTVR with surgical valve repair or replacement. However, in the fall of 2024, the 1-year results of the multicenter, randomized TRISCEND II clinical trial were published. The trial aimed to compare the safety and effectiveness of combining the EVOQUE TTVR system with optimal medical therapy as compared to optimal medical therapy alone in patients with severe TR [86]. The trial included 400 patients and evaluated all patients after 1 year for safety and efficacy. It looked at endpoints such as all-cause mortality, an improvement in NYHA class equal to or greater than 1, an improvement in KCCQ Overall Summary Score (KCCQ-OS) of equal to or greater than 10 points, and an improvement in 6 min walk distance equal to or greater than 30 m. Of the 400 patients (75.5% women), 267 patients were randomized to the TTVR with optimal medical therapy (OMT) group and 133 patients were randomized to the OMT alone group [87]. Eight patients assigned to TTVR with OMT group did not undergo an attempted procedure and were excluded. The trial’s findings are summarized below [87,88,89]. A similar retrospective study, comparing the LuX-Valve with guideline-directed medical therapy (GDMT) versus GDMT alone was published in 2024 [90]. The study (67.7% women), with a follow-up of 20 months, evaluated 31 patients in the interventional group and 57 patients in the control group. The findings from both studies are summarized in Table 5.

Finally, although the indications for TTVR may differ from those of TTVr, the literature comparing the clinical outcomes of these two interventions is scarce. In the novel study by Dershowitz et al., 41% and 59% of the included 61 patients underwent TTVR and TTVr, respectively, with 75% of subjects having a TR classification of less than or equal to severe [91]. The study’s echocardiography results showed that TTVR subjects experienced a greater decline in longitudinal tricuspid annular plane systolic excursion and fractional area change (indicating a decline in RV function) compared to TTVr patients, despite a preserved RV stroke volume, but demonstrated a greater TR reduction, a finding which is positively associated with the reverse remodeling of the RV. The authors further stipulated that the reverse remodeling of the RV was associated with a decreased risk of death, HF hospitalization, or repeated TV intervention.

## 6. Future Directions

It is worth noting the increasing role of artificial intelligence in medicine and its potential applications in interventional cardiology, including in TR management. A review by Chrysostomidis et al. published in March 2024 highlighted ongoing efforts to use 3D printing as a tool for both surgical and transcatheter TR management techniques. These 3D printing models of TV have been successfully used for preoperative planning prior to the placement of commercially available devices, and for creating personalized valves in an attempt to optimize intervention outcomes [92].

The advancements of interventional options for the management of TR, and the promising results regarding their efficacy and safety, in addition to the limited medical options for TR management, open the door to further research in this field. Artificial intelligence (AI) has also started to be considered an important contributor in assessments of valvular disease. A study by Kirchner et al. assessed AI-driven analyses of cardiac computed tomography (CT) images of patients undergoing TTVR. The authors found that they could not only reproduce AI-driven 3D reconstructions of the RV during diastole and systole based on analyzed CT images, but also that AI-facilitated chamber quantification showed an excellent correlation compared to conventional, core-lab evaluated CT analysis [93]. Although AI is not free of biases, its automated segmentation of echocardiographic, CT, and Magnetic Resonance Imaging (MRI) images could aid in fast and accurate structural valve recognition and delineation [94]. As artificial intelligence gains ground in medicine, including in interventional cardiology, the integration of existing surgical and interventional tricuspid valve repair or replacement with 3D printing techniques and machine learning can help advance personalized medicine and lead to further research on this matter in an unrelenting endeavor to treat illness and achieve better outcomes.

## 7. Conclusions

As previously mentioned, there are currently no clinical trials comparing heterotopic TTVR systems or the long-term clinical outcomes of heterotopic with orthotopic TTVR systems. At the same time, the literature comparing the clinical outcomes between transcatheter and surgical repair or replacement systems is scarce. With a plethora of new technologies, further research should compare the safety and efficacy of current and emerging state-of-the-art innovations. The promising results of the newly approved EVOQUE and TriClip systems may thus propel transcatheter repair and replacement as the standard recommended first-line treatment for severe TR in conjunction with medical therapy. Nonetheless, the final decision regarding the treatment of TR should remain patient-centered, taking into consideration the hemodynamic stability and surgical risk for each individual.

## Figures and Tables

**Table 1:** Retrospective studies comparing medical therapy with surgical intervention for severe tricuspid regurgitation (TR). CKD: Chronic Kidney Disease; HF: Heart Failure; OS: Overall Survival; HR: Hazard Ratio; CI: Confidence Interval; p: *p*-Value.

Retrospective Studies on Medical Therapy vs. Surgical Intervention for Severe TR	
Axtell et al. [65]	1. Retrospective study of 3276 patients with isolated TR; 171 underwent surgical intervention, with 3105 remaining medically managed. 2. No statistical difference in the OS among the control and surgical intervention groups (HR 1.34 95%CI 0.78–2.30, *p* = 0.288). 3. Subjects who were offered surgery were younger in age and were less likely to suffer from comorbidities such as CKD, diabetes, and HF than patients who were medically managed; the comorbidities were not statistically significant among these two groups.
Wang et al. [66]	1. Retrospective study of 9031 patients with isolated TR graded as belonging to the moderate–severe category. A total of 632 underwent surgical intervention. 2. Surgery patients exhibited statistically significant greater OS rates of 86% and 69% at 1 and 5 years, respectively, compared to the 71% and 46% rates observed for the medically managed group for the same period of time. 3. Tricuspid surgery patients had significantly greater rates of infective endocarditis (HR 5.55 95%CI 4–7.71) and HF hospitalizations (HR 1.29 95%CI 1.16–1.43), but similar rates for stroke (HR 1.09 95%CI 0.76–1.57) and myocardial infarctions (HR 1.06 95%CI 0.77–1.47) to those that were medically managed. They were also less likely to have comorbidities.
Dreyfus et al. [67]	1. Retrospective study of 1768 patients with severe isolated functional TR 551 that underwent surgical intervention. Subjects were almost equally divided in thirds according to TRI-SCORE categories (33%; 32%; 35%). 2. The inverse propensity weighted survival rates at 10 years were higher in both the repair (HR 0.11 95%CI 0.06–0.19) and replacement (HR 0.65; 95%CI 0.47–0.90) groups. compared with the conservative management group in the low-TRI-SCORE category 3. The repair group exhibited a higher survival rate compared to the conservative group (HR 0.49 95%CI 0.35–0.68) in the intermediate-TRI-SCORE category. 4. The replacement group was considered possibly harmful in both the intermediate- and high-TRI-SCORE categories (HR of 1.43 and 1.58, respectively). 5. Survival was higher in the repair than in the replacement group among all TRI-SCORE categories.

**Table 2:** Table summarizing the findings of the TRILUMINATE and TRILUMINATE Pivotal studies. TV: Tricuspid Valve; TR: Tricuspid Regurgitation; HF: Heart Failure; NYHA: New York Heart Association; KCCQ: Kansas City Cardiomyopathy Questionnaire; TTVR: Transcatheter Tricuspid Valve Replacement.

Findings of TRILUMINATE Study and TRILUMINATE Pivotal Trial	
TRILUMINATE Study [69]	Three years after the procedure, 98 subjects exhibited the following: 1. TR reduction to moderate or less in 92% of patients. 2. TR reduction by 1 grade or more in 92% of patients. 3. NYHA class improvement, with 79% of patients at class III/IV at baseline vs. 19% of patients at class III/IV at the endpoint. 4. KCCQ score decreased by a mean of 10 ± 3 points between baseline and endpoint. 5. Reduction in all-cause hospitalizations by 53%. 6. Reduction in HF hospitalizations by 75%.
TRILUMINATE Pivotal Trial [70]	A total of 350 patients were enrolled, 175 of which were in the control group. At year 1 after procedure, the following observations were made: 1. Survivability and lack of need for TV surgery: 90.6% in TriClip group vs. 89.9% in control group (*p* = 0.82). 2. Rate of HF hospitalization: 17% in TriClip group vs. 20% in control group (*p* = 0.40). 3. KCCQ score improvement of ≥15-point: 52.3% in TriClip group vs. 23.5% in control group (*p* < 0.0001). 4. Overall KCCQ score change: 13.0 ± 1.4 points in TriClip group vs. −0.5 ± 1.4 points in control group (*p* < 0.0001). 5. Six-minute walk distance change: 1.7 ± 7.5 m in TriClip group vs. −27.4 ± 7.4 m in control group (*p* < 0.0001). 6. Lack of major adverse events: 89.9% in TriClip group vs. 90% performance goal (*p* < 0.0001).

**Table 3:** Table summarizing clinical trials studying experimental TTVr devices. TTVr: Transcatheter Tricuspid Valve Repair; TV: Tricuspid Valve; TR: Tricuspid Regurgitation; NYHA: New York Heart Association; KCCQ: Kansas City Cardiomyopathy Questionnaire.

Findings of Clinical Trials and Studies Evaluating TTVr Interventions	
CLASP TR—PASCAL system [41]	Sixty-five patients were enrolled. At 1 year after procedure: 1. TR was reduced to moderate or less in 86% of patients. 2. TR was reduced by 1 grade or more in all patients. 3. An NYHA functional class improvement was observed, with 70.8% of patients at class III/IV at baseline vs. 92% of patients at class I/II at endpoint. 4. KCCQ score improvement by 18 points. 5. Survivability: 87.9% of patients. 6. Freedom from hospitalization for HF: 78% of patients.
The Edwards Cardioband Tricuspid Valve Reconstruction System Early Feasibility study [73]	Thirty-seven patients enrolled. At 1 year after procedure: 1. TR reduction to moderate or less in 73% of patients. 2. TR reduction by 2 or more grades in 73.1% of paatients. 3. NYHA functional class improvement, with 65% of patients at class III/IV at baseline vs. 92% of patients at class I/II at endpoint. 4. KCCQ score improvement by 19 points. 5. Survivability rate: 85.9%. 6. Freedom from hospitalization for HF: 88.7%.
TRI-REPAIR study—Edwards Cardioband system [74]	Thirty patients were enrolled. At 2 years after procedure: 1. TR reduction to moderate or less in 72% of patients. 2. NYHA functional class improvement with 83% of patients at class III/IV at baseline vs. 82% at class I/II at endpoint. 3. Improvement in 6 min walk distance by 73 m. 4. KCCQ score improvement by 14 points. 5. Survivabilityrate: 73 ± 8%. 6. Freedom from hospitalization for HF: 56 ± 10%.
MATTERS and MATTERS II trial—Mistral technique [75]	Nine patients were enrolled. At 1 year after procedure: 1. TR severity reduction from baseline of 33.3% of patients with severe TR, 55.5% of patients with massive TR, and 11.1% of patients with torrential TR to 56% of patients with mild TR and 44% with severe TR at endpoint. 2. TR reduction by at least 1 grade in 100% of patients. 3. NYHA functional class improvement with 100% of patients at class III at baseline vs. 75% of patients at class I/II at endpoint. 4. Improvement in 6 min walk distance by +105.14 m. 5. KCCQ score improvement by 22.55 points. 6. Survivability: 100%.
TriStar trial—K-clip [76]	Ninety-six patients were enrolled. At 1 year after procedure: 1. Reduction in TR by at least 1 grade in 94.2% of patients and at least 2 grades in 87.2% of patients. 2. NYHA improvement, with 97.7% at class I/II at endpoint. 3. KCCQ score improvement by 7 points. 4. Survivability: 94.7% ± 2.3%. 5. Freedom from hospitalization for HF: 90.4% ± 3.0%.

**Table 4:** Reported outcomes after 1 year for the TricValve and TRICENTO systems. NYHA: New York Heart Association; KCCQ: Kansas City Cardiomyopathy Questionnaire; HF: Heart Failure; PPM: Permanent Pacemaker.

1-Year Outcomes for the TricValve and TRICENTO Systems	
TricValve [85]	Forty-four patients were included. The following findings were observed: A total of 62.2% of patients advanced to an NYHA functional class I or II. An HF rehospitalization rate of 29.5% and all-cause mortality rate of 6.8%. No significant improvement was detected in the 6 min walk test, but a significant improvement in the overall quality of life of at least 15 points was detected using a KCCQ-12 score. A total of 9% of patients experienced gastrointestinal hemorrhages and a stroke during follow-up. Hepatic vein backflow was abolished in 63.8% of patients, but no significant change was seen in hepatic and renal function. A total of 40% of the patients maintained a low dose of loop diuretics.
TRICENTO [54]	Twenty-one patients were enrolled. The following findings were observed: Overall survival rate of 76%, with a hospitalization for right-sided HF percentage of 19%. No cases of new PPM implantation were reported. A total of 65% of patients were assigned to an NYHA functional class I or II, but no significant change was observed in cardiac output. No major bleeding, cerebrovascular incident, or myocardial infarction were observed in any of the patients at follow-up. No significant change was seen in hepatic and renal function. The dosage of diuretics was reduced in 61% of patients.

**Table 5:** Table summarizing the findings of the TRISCEND II and LuX-Valve clinical trials. HF: heart failure; PPM: permanent pacemaker; GDMT: guideline-directed medical therapy; OMT: optimal medical therapy; KCCQ-OS: Kansas City Cardiomyopathy Questionnaire Overall Summary; TTVR: Transcatheter Tricuspid Valve Replacement.

Findings of the TRISCEND II and LuX-Valve Clinical Trials	
TRISCEND II trial [87,88,89]	A total of 392 patients, of whom 259 underwent attempted TTVR and 133 received OMT alone. The following findings were observed: 1. No significant differences in all-cause mortality (12.6% vs. 15.2%), and HF hospitalization (20.9% vs. 26.1%) among these two groups (TTVR+OMT vs. OMT alone). 2. Mean between-group differences in the KCCQ-OS favored the TTVR+OMT group at 30 days (11.8 points), 6 months (20.8 points), and 1 year (17.8 points). 3. Favorable win ratio for the TTVR+OMT group (10.2) compared to OMT alone (0.8) regarding improvement in NYHA Functional Class. 4. Favorable win ratio for the TTVR+OMT group (1.1) compared to OMT alone (0.9) regarding improvement in 6 min walk test. 5. Greater incidences of bleeding and new PPM implantation in the TTVR+OMT group (15.4% and 17.4%) compared to the OMT-alone group (5.3% and 2.3%). 6. No patients in either group underwent the implantation of a right ventricular assist device or heart transplantation. 7. Fewer wins for the TTVR+OMT group (win ratio of 9.7) compared to OMT alone (win ratio of 10) regarding annualized rate of hospitalization for HF. 8. The Kaplan–Meier estimates for postindex tricuspid-valve intervention were 13.7 ± 2.2% and 20.8 ± 3.7% for the interventional and control group, respectively.
Randomized Trial comparing LuX-Valve with GDMT versus GDMT alone [90]	A total of 88 patients were included, 57 of them receiving GDMT alone and 31 of them receiving combined TAVR and GDMT. The following findings were observed: 1. Superior 2-year survival rate in the interventional group (75.8%) when compared to the control group (48.4%). 2. Significant freedom from combined endpoint (all-cause mortality and HF hospitalization) at 2 years for the interventional group (61.5%) compared to the control group (45.9%). 3. Patients in the interventional group experienced a statistically significant decrease in the RV mid diameter, and substantial increases in the six-minute walk test, KCCQ and NYHA functional class at the 6-month follow-up. 4. Regarding major adverse events that were statistically different among groups, there was a greater risk for gastrointestinal hemorrhage and renal failure requiring dialysis in the control group.
